# Au_23_(CR)_14_ nanocluster restores fibril Aβ’s unfolded state with abolished cytotoxicity and dissolves endogenous Aβ plaques

**DOI:** 10.1093/nsr/nwz215

**Published:** 2019-12-20

**Authors:** Wenkang Zhang, Guanbin Gao, Zhongjie Ma, Zhuoying Luo, Meng He, Taolei Sun

**Affiliations:** 1 State Key Laboratory of Advanced Technology for Materials Synthesis and Processing, Wuhan University of Technology, Wuhan 430070, China; 2 School of Chemistry, Chemical Engineering and Life Science, Wuhan University of Technology, Wuhan 430070, China

**Keywords:** gold nanoclusters, Alzheimer's disease, restores fibril Aβ’s unfolded state, abolished cytotoxicity, dissolves endogenous Aβ plaques

## Abstract

The misfolding of amyloid-β (Aβ) peptides from the natural unfolded state to β-sheet structure is a critical step, leading to abnormal fibrillation and formation of endogenous Aβ plaques in Alzheimer's disease (AD). Previous studies have reported inhibition of Aβ fibrillation or disassembly of exogenous Aβ fibrils *in vitro*. However, soluble Aβ oligomers have been reported with increased cytotoxicity; this might partly explain why current clinical trials targeting disassembly of Aβ fibrils by anti-Aβ antibodies have failed so far. Here we show that Au_23_(CR)_14_ (a new Au nanocluster modified by Cys-Arg (CR) dipeptide) is able to completely dissolve exogenous mature Aβ fibrils into monomers and restore the natural unfolded state of Aβ peptides from misfolded β-sheets. Furthermore, the cytotoxicity of Aβ_40_ fibrils when dissolved by Au_23_(CR)_14_ is fully abolished. More importantly, Au_23_(CR)_14_ is able to completely dissolve endogenous Aβ plaques in brain slices from transgenic AD model mice. In addition, Au_23_(CR)_14_ has good biocompatibility and infiltration ability across the blood–brain barrier. Taken together, this work presents a promising therapeutics candidate for AD treatment, and manifests the potential of nanotechnological approaches in the development of nanomedicines.

## Teaser

Au_23_(CR)_14_, a Cys-Arg (CR) dipeptide modified gold nanocluster, restores fibril Aβ's unfolded state with abolished cytotoxicity and dissolves endogenous Aβ plaques, promising for Alzheimer's disease treatment.

## INTRODUCTION

A hallmark sequence of events in Alzheimer's disease (AD) is the misfolding, fibrillation and accumulation of amyloid-β (Aβ) peptides, resulting in cellular dysfunction, loss of synaptic connections and brain damage [1–4]. Over the past three decades, the inhibition of Aβ fibrillation and the disassembly of deposited Aβ fibrils have been the magnets for searching promising therapeutics for AD treatment [5–9]. A number of inhibitors (including β- and γ-secretase inhibitors) for inhibiting Aβ production were discontinued in phase ii or iii clinical trials due to their low efficacy and serious side effects [10]. Anti-Aβ antibody-based immunotherapy for disassembling the mature Aβ fibrils was once expected to be the first radical treatment of AD [11]. However, prior studies have indicated that the soluble Aβ oligomers, as the most toxic species, might reappear during the disassembly process to induce more neurotoxicity (i.e. the ‘dust-raising’ effect) [12–16]. One approach to ameliorate the toxicity of soluble Aβ oligomers is to promote their aggregation by, for example, chiral silica nanoribbons and star-shaped poly(2-hydroxyethyl acrylate) nanostructures [17,18]. Also, graphene quantum dots are reported to drive the peptide fibrillization off-pathway to eliminate the toxic intermediates, which points to the potential of using zero-dimensional nanomaterials for *in vivo* mitigation of a range of amyloidosis types [19]. Recently, polymer-peptide conjugates and curcumin–gold nanoparticles (AuNPs) with hydrodynamic diameters of 10–25 nm have been shown to disassemble exogenous Aβ fibrils *in vitro*, but they failed to restore the natural unfolded state of Aβ from the misfolded β-sheets [20–22]. However, the β-lactoglobulin ‘coronae’ of the AuNPs are reported to enable X-ray destruction of islet amyloid polypeptide (IAPP) amyloids, providing a viable new nanotechnology against amyloidogenesis [23]. The small molecule epigallocatechin gallate (EGCG) presents the capability to prevent aggregation and remodel amyloid fibrils, which could also convert mature amyloid fibrils to amorphous protein aggregates that are less toxic to cells, implying the possibility of reducing the toxicity of amyloid fibrils by remodeling their molecular structures [24–26]. Therefore, the treatment of AD needs to explore new materials that are able to dissolve endogenous Aβ plaques and abolish the proteotoxicity of Aβ fibrils by restoring their natural unfolded state from the misfolded β-sheets.

To date, nanomaterials and multifunctional nanocomposites possessing certain structural and physicochemical traits are promising candidates for mitigating amyloidosis *in vitro* and *in vivo*, indicating the use of nanoparticles as an emerging field against amyloid diseases [16,27]. Gold nanoclusters (AuNCs) (*d* < 3 nm) have been widely studied in biomedical fields due to their small-size effect and good biocompatibility [28–30]. Our previous study has shown that glutathione-modified AuNCs (GSH-AuNCs) can completely inhibit the fibrillation of Aβ peptides [31,32]. This study was expanded to explore whether any AuNCs including GSH-AuNCs could dissolve mature exogenous Aβ fibrils and endogenous Aβ senile plaques, and, more importantly, restore the natural unfolded state of Aβ from the misfolded β-sheets. To this end, seven kinds of AuNCs (i.e. Cys-AuNCs, CSH-AuNCs, *p*-MBA-AuNCs, MPA-AuNCs, GSH-AuNCs, NIBC-AuNCs and CR-AuNCs) modified by cysteine, cysteamine, 4-mercaptobenzoic acid, mercaptopropionic acid, glutathione, *N*-isobutyryl-*L*-cysteine or Cys-Arg, respectively, have been synthesized.

## RESULTS AND DISCUSSION

### Seven kinds of AuNCs on inhibiting A**β** fibrillation

**Figure 1.**
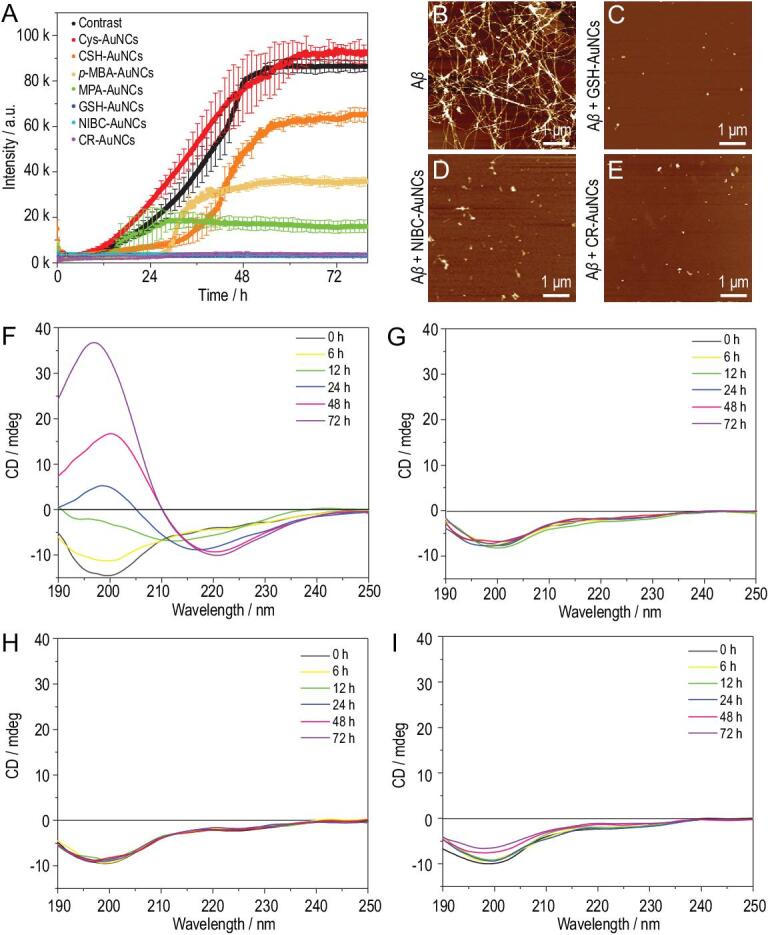
(A) Fibrillation kinetics for 20 μmol·L^−1^ Aβ_40_ in the absence (black) or presence of 25 mg·L^−1^ Cys-AuNCs, CSH-AuNCs, *p*-MBA-AuNCs, MPA-AuNCs, GSH-AuNCs, NIBC-AuNCs or CR-AuNCs. (B–E) AFM images of Aβ_40_ after 72 h co-incubation in the absence (B) or presence of 25 mg·L^−1^ GSH-AuNCs (C), NIBC-AuNCs (D) and CR-AuNCs (E). (F–I) *In situ* real-time CD spectra monitoring of 20 μmol·L^−1^ Aβ_40_ peptides co-incubated without (F) or with 25 mg·L^−1^ GSH-AuNCs (G), NIBC-AuNCs (H) and CR-AuNCs (I) for 72 h.

First, the effects of these seven kinds of AuNCs on inhibiting Aβ fibrillation were investigated by co-incubating 20 μmol·L^−1^ Aβ_40_ with each kind of AuNCs at the same concentration (25 mg·L^−1^). The concentrations were selected based on their solubility and biological relevance from our preliminary experiments. The standard thioflavine-T (ThT) binding fluorescence assay was employed to record the fibrillation kinetics. As shown in Fig. 1A, the fibrillation kinetics of 20 μmol·L^−1^ Aβ_40_ without AuNCs showed a standard S-curve (black curve); the formation of preformed/mature Aβ_40_ fibrils was confirmed by atomic force microscopy (AFM) images (Fig. 1B; Fig. S1 in the online supplementary material). Cys-AuNCs had no inhibitory effect (red curve); CSH-AuNCs (orange curve), *p*-MBA-AuNCs (yellow curve) and MPA-AuNCs (green curve) showed partial inhibition. Consistent with our previous studies, GSH-AuNCs showed complete inhibition of Aβ_40_ fibrillation (blue curve). Encouragingly, NIBC-AuNCs (cyan curve) and CR-AuNCs (purple curve) were also able to completely inhibit Aβ_40_ fibrillation, which was further verified by AFM images (no fibrils could be found in Fig. 1C–E). Moreover, *in situ* real-time circular dichroism (CD) spectra were used to record the conformational transition of Aβ_40_ in the fibrillation process. As shown in Fig. 1F, in the absence of AuNCs, Aβ_40_ had undergone a misfolding process from an unfolded state (negative peak at 198 nm) into a β-sheet structure (negative peak at 220 nm). Interestingly, GSH-AuNCs (Fig. 1G), NIBC-AuNCs (Fig. 1H) and CR-AuNCs (Fig. 1I) could maintain the unfolded state of Aβ_40_ peptides throughout the incubation. It should be noted that the seven AuNCs used have similar particle sizes (1.6 ± 0.5 nm), and their transmission electron microscope (TEM) images and the UV-visible absorption spectra are shown in Fig. S2.

### Seven kinds of AuNCs on the dissolving of mature A**β** fibrils

**Figure 2.**
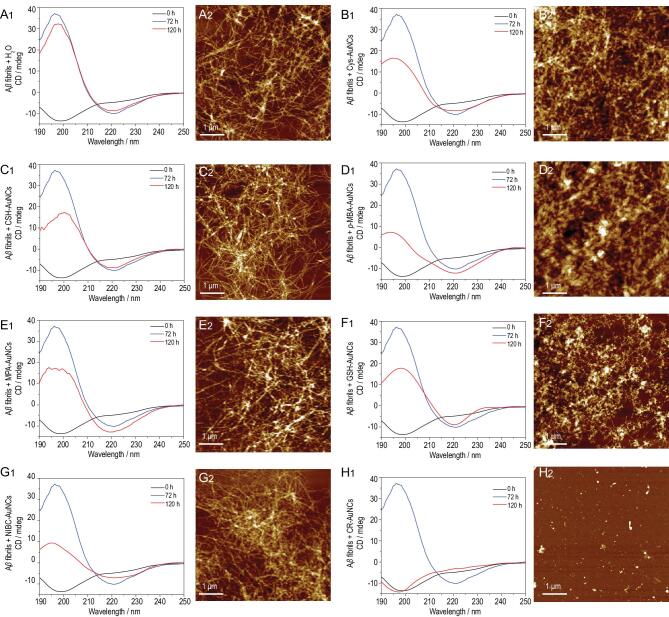
(A_1_–H_1_) CD spectra of 20 μmol·L^−1^ Aβ_40_: black curves (0 h) denoting 0 h prior to incubation; blue curves (72 h) denoting pre-incubation for 72 h; red curves (120 h) denoting the co-incubation for 48 h of preformed Aβ_40_ fibrils in the (A_1_) absence or presence of 50 mg·L^−1^ (B_1_) Cys-AuNCs, (C_1_) CSH-AuNCs, (D_1_) *p*-MBA-AuNCs, (E_1_) MPA-AuNCs, (F_1_) GSH-AuNCs, (G_1_) NIBC-AuNCs and (H_1_) CR-AuNCs. (A_2_–H_2_) AFM images of the samples at the end of CD experiments (120 h): (A_2_) blank control, (B_2_) Cys-AuNCs, (C_2_) CSH-AuNCs, (D_2_) *p*-MBA-AuNCs, (E_2_) MPA-AuNCs, (F_2_) GSH-AuNCs, (G_2_) NIBC-AuNCs and (H_2_) CR-AuNCs.

Since inhibition of fibrillation and dissolution of fibrils should be considered as two discrete events, then whether these AuNCs could dissolve preformed/mature Aβ_40_ fibrils was investigated by using CD and AFM. Freshly prepared Aβ_40_ (20 μmol·L^−1^) were pre-incubated at 37°C for 72 h. The preformed Aβ_40_ fibrils were then co-incubated with 50 mg·L^−1^ individual AuNCs for 48 h. The formation and dissolution of Aβ_40_ fibrils were recorded by CD. The data showed that the peak at 220 nm did not change between 72 h and 120 h when treated with Cys-AuNCs (Fig. 2B_1_), CSH-AuNCs (Fig. 2C_1_), *p*-MBA-AuNCs (Fig. 2D_1_), MPA-AuNCs (Fig. 2E_1_), GSH-AuNCs (Fig. 2F_1_) and NIBC-AuNCs (Fig. 2G_1_), and that the fibrils were intact (Fig. 2B_2_–G_2_), indicating no dissolution of the mature Aβ_40_ fibrils. The failure of GSH-AuNCs to dissolve Aβ_40_ fibrils confirmed that inhibition of fibrillation and dissolution of fibrils are two discrete events. Most excitingly, when treated by CR-AuNCs, the peak at 220 nm (i.e. β-sheet) disappeared and the peak at 198 nm (i.e. unfolded state) resurfaced (Fig. 2H_1_). The dissolution of the mature Aβ_40_ fibrils by CR-AuNCs was further confirmed by AFM observation (Fig. 2H_2_). The perfect overlay of CD curves of 0 h and 120 h demonstrated that CR-AuNCs could completely dissolve the mature Aβ_40_ fibrils, and fully restore the unfolded state of Aβ_40_ peptides from β-sheet structure.

### Molecular composition and structure of CR-AuNCs

**Figure 3.**
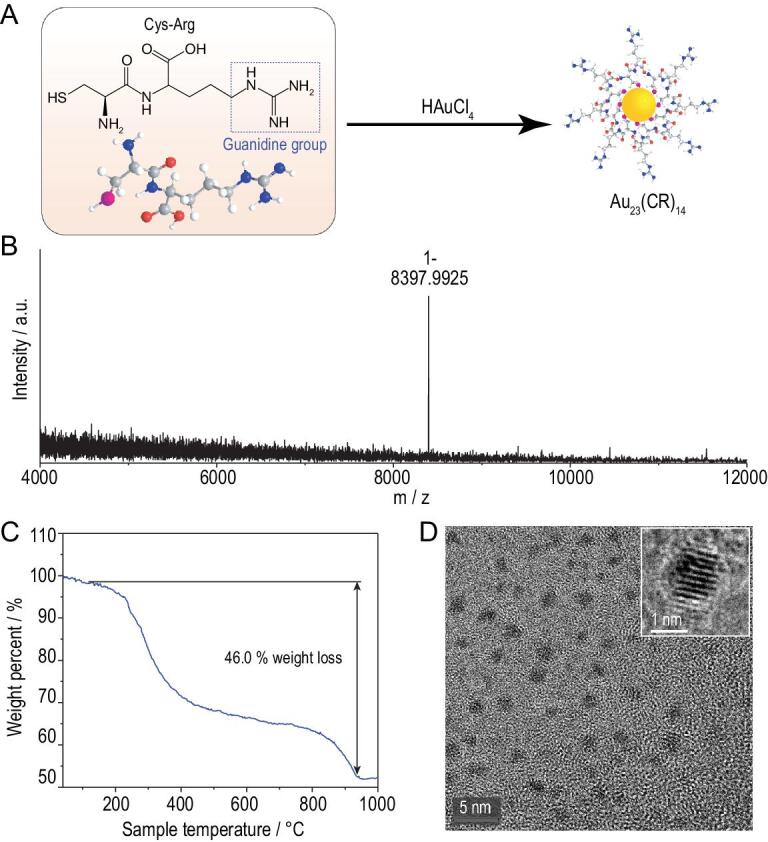
(A) Scheme of synthesis of CR-AuNCs and characterization of CR-AuNCs by (B) ESI-MS analysis, (C) thermogravimetric analysis and (D) TEM image.

To ascertain their molecular composition and structure, CR-AuNCs were characterized using various technical platforms (Fig. 3 and Fig. S3). The electrospray ionization mass spectrometry (ESI-MS) analysis showed a single distinct peak at 8397.9925, indicating that CR-AuNCs had a formula of Au_23_(CR)_14_ (Fig. 3A). The formula was further confirmed by thermogravimetric analysis (Fig. 3B). The weight loss of 46.0% meant that the CR weight ratio agrees well with the formula of Au_23_(CR)_14_ (calculated loss: 46.0%). In addition, the high resolution TEM analysis showed that the Au_23_(CR)_14_ had a spherical morphology (Fig. 3C), where the shape was regular with a clear lattice fringe (inset of Fig. 3C).

### The process detail and possible mechanisms of Au_23_(CR)_14_ dissolving the preformed A**β**_40_ fibrils

**Figure 4.**
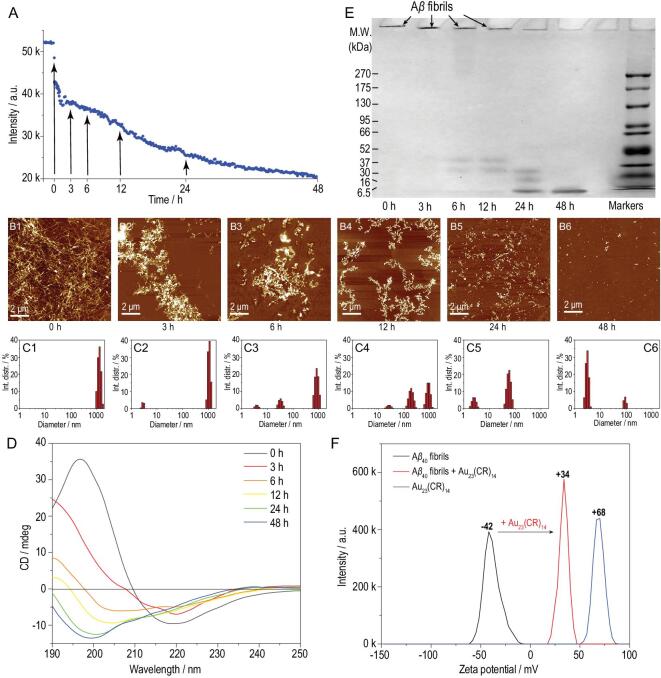
(A) The kinetics of Aβ_40_ fibrils co-incubated with 50 mg·L^−1^ Au_23_(CR)_14_ for 48 h. (B) AFM images of Aβ_40_ fibrils co-incubated with 50 mg·L^−1^ Au_23_(CR)_14_ for (B_1_) 0 h, (B_2_) 3 h, (B_3_) 6 h, (B_4_) 12 h, (B_5_) 24 h and (B_6_) 48 h. (C) DLS results of Aβ_40_ fibrils co-incubated with 50 mg·L^−1^ Au_23_(CR)_14_ for (C_1_) 0 h, (C_2_) 3 h, (C_3_) 6 h, (C_4_) 12 h, (C_5_) 24 h and (C_6_) 48 h. (D) *In situ* real-time CD spectra of 20 μmol·L^−1^ Aβ_40_ fibrils co-incubated with 50 mg·L^−1^ Au_23_(CR)_14_. (E) Native PAGE gel electrophoresis analysis of Au_23_(CR)_14_-treated preformed Aβ_40_ fibrils for designated times. (F) Zeta potential of preformed Aβ_40_ fibrils immediately after the injection of Au_23_(CR)_14_.

To gain more insights into the dissolution process, preformed Aβ_40_ fibrils were co-incubated with 50 mg·L^−1^ Au_23_(CR)_14_ for 48 h. The dissolution dynamics were monitored by ThT assay. The fluorescence intensity declined continuously during 48 h incubation (Fig. 4A), indicating a gradual process of dissolution. The gradual dissolution of Aβ_40_ fibrils had also been evidenced by AFM studies (Fig. 4B_1_–B_6_). The apparent sizes of the samples were assayed by dynamic light scattering (DLS). The DLS results showed that the apparent sizes of the samples decreased from over 1000 nm to less than 10 nm (Fig. 4C_1_–C_6_). The *in situ* real-time CD spectra revealed that the peak at 220 nm was continuously shifted to 198 nm (Fig. 4D), indicating that the dissolution of Aβ_40_ fibrils by Au_23_(CR)_14_ is a dynamic process accompanied by a conformational transition from a β-sheet structure to an unfolded state. The native PAGE results showed one band with a molecular weight less than 6.5 kDa (Fig. 4E, 48 h), directly demonstrating that Au_23_(CR)_14_ completely dissolves Aβ_40_ fibrils into monomers (∼4.2 kDa).

To explore possible mechanisms of how Au_23_(CR)_14_, but not the other six kinds of AuNCs, could dissolve Aβ_40_ fibrils, the zeta potentials of Aβ_40_ fibrils, individual AuNCs and Aβ_40_ fibrils, together with individual AuNCs, were measured. The median of the zeta potential of mature Aβ_40_ fibrils was −41 ± 2 mV (black curves in Fig. 4F and Fig. S4). Cys-AuNCs, CSH-AuNCs, *p*-MBA-AuNCs, MPA-AuNCs, GSH-AuNCs, NIBC-AuNCs and Au_23_(CR)_14_ have a zeta potential of −32, +36, −49, −57, +2, −34 and +68 mV, respectively (blue curves in Fig. 4F and Fig. S4). After addition of AuNCs, while the mixtures with the other six kinds of AuNCs showed a negative zeta potential with a median value from −44 to −18 mV, the mixture with Au_23_(CR)_14_ showed a positive zeta potential with a median value of +34 mV (red curves in Fig. 4F and Fig. S4). These data suggest that Au_23_(CR)_14_ adsorb onto Aβ_40_ fibrils more strongly than other AuNCs. In consideration of Aβ_40_ monomers with a net charge of negative 2.7 at physiological pH (7.4) [33], and the existence of a guanidine group in the residue of CR that could be protonated in a wide range of pH [34], the strong electrostatic interaction between Aβ_40_ and Au_23_(CR)_14_ might drive the gradual dissolution of mature Aβ_40_ fibrils. The above results strongly suggest that Au_23_(CR)_14_ dissolve the preformed/mature Aβ_40_ fibrils from misfolded β-sheets into the unfolded monomer state through strong electrostatic interactions.

### Au_23_(CR)_14_-mediated A**β**_40_ fibril dissolution on cell viabilities

**Figure 5.**
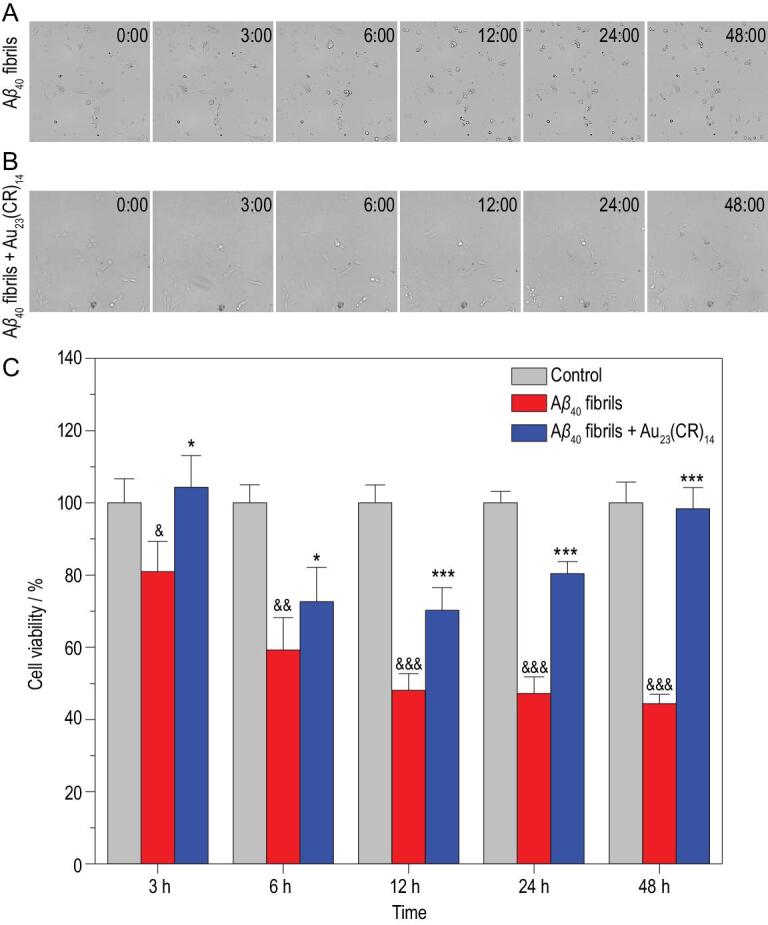
(A, B) *In situ* real-time morphological changes of PC-12 cells co-incubated with freshly preformed Aβ_40_ fibrils without (A) or with 50 mg·L^−1^ Au_23_(CR)_14_ (B). (C) Viabilities of PC-12 cells after Aβ_40_ fibril treatment in the absence (red) and presence (blue) of 50 mg·L^−1^ Au_23_(CR)_14_ for 3, 6, 12, 24 and 48 h. No treatment was included as the blank control (gray). Student's *t*-test: *n* = 5, ^*^P < 0.05, ^***^P < 0.001 vs. Aβ_40_ fibrils-induced group; ^&^*P* < 0.05, ^&&^*P* < 0.01, ^&&&^*P* < 0.001 vs. control.

To investigate the effect of Au_23_(CR)_14_-mediated dissolution of Aβ_40_ fibrils on cell viabilities, an AD cell model based on Aβ_40_ fibril-induced cell deaths of PC-12 cells was adopted [35]. First, PC-12 cells were co-incubated with freshly preformed Aβ_40_ fibrils without (Fig. 5A) or with (Fig. 5B) Au_23_(CR)_14_, and *in situ* real-time morphological changes were recorded by a Cytation 5 Cell Imaging Multi-Mode Reader. Aβ_40_ fibrils formed from 20 μmol·L^−1^ monomers were used to cause a 50% decrease of cell viability based on our preliminary titration experiments. As shown in Fig. 5A, when treated with Aβ_40_ fibrils alone, cell shrinkage started to appear in the 3rd hour, and then cells with reduced sizes and round shapes apparently increased from the 12th to the 48th hour. In contrast, when PC-12 cells were treated with Aβ_40_ fibrils and 50 mg·L^−1^ Au_23_(CR)_14_, no obvious morphological changes were observed (Fig. 5B). The corresponding videos are shown in the online supplementary material. Second, a CCK-8 assay was used for quantifying cell viabilities. Freshly preformed Aβ_40_ fibrils from 20 μmol·L^−1^ monomers were added into PC-12 cells with or without Au_23_(CR)_14_; the cells were cultured and sampled at the 3rd, 6th, 12th, 24th and 48th hour for assaying their viabilities. No treatment was included as the blank control. As shown in Fig. 5C, the cell viability was not affected in the blank control group (gray bars), and the addition of preformed Aβ_40_ fibrils alone caused a gradual decrease of cell viability to 50% (red bars). In contrast, when cells were cultured with 50 mg·L^−1^ Au_23_(CR)_14_ together with preformed Aβ_40_ fibrils, the cell viability decreased initially to 70% at the 12th hour and then started to increase, reaching almost 100% (same as the blank control) at the 48th hour (blue bars). These data collectively demonstrated that Au_23_(CR)_14_ could fully abolish the cytotoxicity of Aβ_40_ fibrils. As for the two phasic characteristics of cell viabilities in the Au_23_(CR)_14_ treatment, we speculate that the toxic oligomers [36,37] were produced during the dissolution process and the cytotoxicity was fully abolished when the oligomers were completely dissolved into non-toxic monomers.

### The capacity of Au_23_(CR)_14_ for dissolving exogenous A**β** fibrils

**Figure 6.**
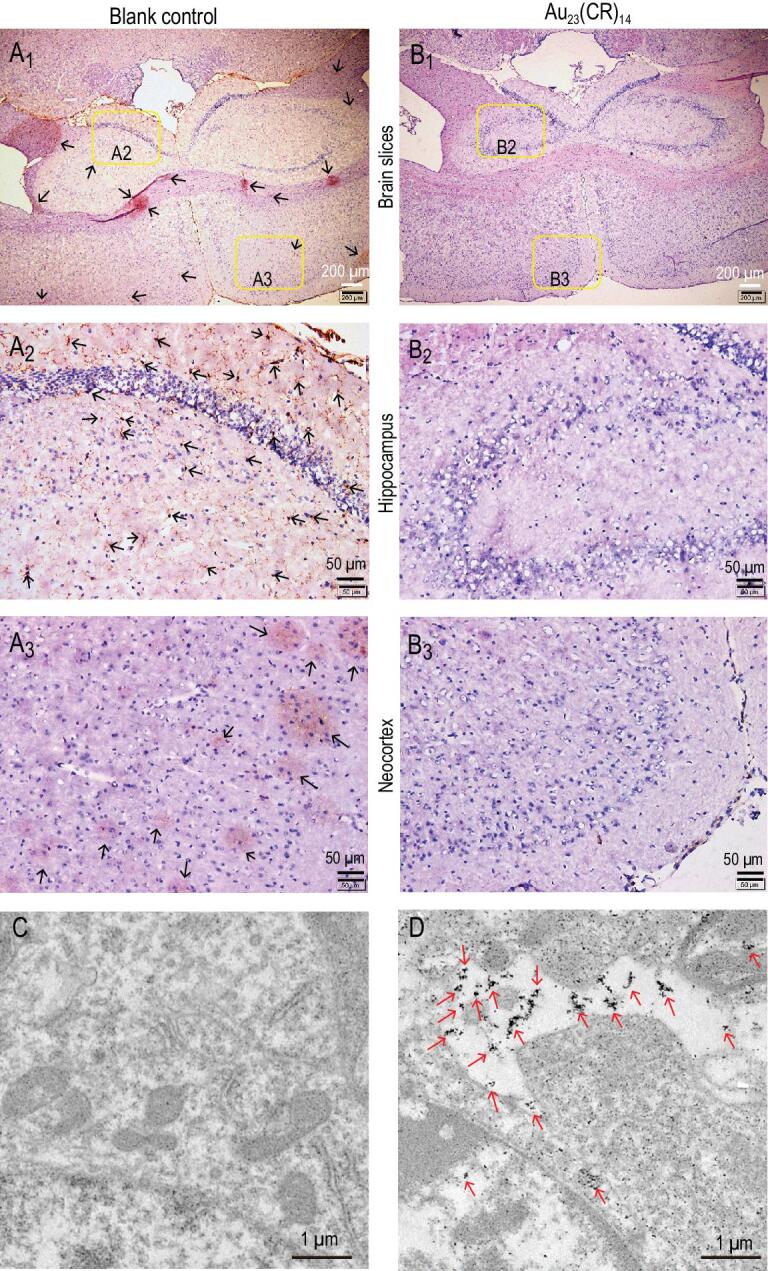
Immunohistochemical analyses of the hippocampus and the neocortex in the brain slices of AD model mice. The brain slices were co-incubated without (A) or with (B) 50 mg·L^−1^ Au_23_(CR)_14_ for 24 h after fixing on the glass slides. (A2) and (B2) are the partial enlargement of hippocampus. (A3) and (B3) are the partial enlargement of neocortex. (C, D) TEM images of the mouse slices at 6 h after intraperitoneal injection of Au_23_(CR)_14_ with a dose of 20 mg·kg^−1^ (D) or same volume of normal saline (C) in the normal mice. The presence of Au_23_(CR)_14_ is marked by red arrows.

The ultimate test is whether the capacity of Au_23_(CR)_14_ for dissolving exogenous Aβ fibrils can be translated into dissolving the endogenous Aβ plaques. We obtained brain slices derived from the resected brain tissue of an adult transgenic mouse model of AD, where the brain slices contained endogenous Aβ plaques. The brain slices were co-incubated without (Fig. 6A_1_–A_3_) or with (Fig. 6B_1_–B_3_) Au_23_(CR)_14_ for 24 h, and then the slices were stained with anti-Aβ antibodies for immunohistochemical analyses. Figure 6A_1_–A_3_ shows that the hippocampus and the neocortex were present with a large amount of endogenous Aβ plaques (yellow-brown patches indicated by the arrows). Excitingly, the treatment with 50 mg·L^−1^ Au_23_(CR)_14_ eliminated all yellow-brown patches (Fig. 6B_1_–B_3_), demonstrating that Au_23_(CR)_14_ could completely dissolve the endogenous Aβ plaques in the hippocampus and the neocortex. Furthermore, our data showed that Au_23_(CR)_14_ did not affect cell viability at a concentration of as high as 100 mg·L^−1^ (Fig. S5), indicating good biocompatibility. In addition, the overcoming of the blood–brain barrier is one precondition of nanomaterials in treating neurological diseases [6]. Our data showed that Au_23_(CR)_14_ particles were readily detected in the brain tissues when intraperitoneally injected into normal mice, demonstrating that Au_23_(CR)_14_ is capable of overcoming the blood–brain barrier (Fig. 6D).

## CONCLUSION

In conclusion, seven kinds of AuNCs (i.e. Cys-AuNCs, CSH-AuNCs, *p*-MBA-AuNCs, MPA-AuNCs, GSH-AuNCs, NIBC-AuNCs and Au_23_(CR)_14_) were synthesized and adopted to investigate their effects on the dissolution of mature Aβ fibrils and the restoration of the unfolded state of Aβ peptides. Among the seven kinds of AuNCs tested, only Au_23_(CR)_14_ are able to completely dissolve exogenous mature Aβ fibrils into monomers, and fully abolish cytotoxicity by restoring the natural unfolded state of Aβ peptides from misfolded β-sheets. Furthermore, Au_23_(CR)_14_ are able to completely dissolve endogenous Aβ plaques in the brain slices from transgenic AD model mice. In addition, Au_23_(CR)_14_ have good biocompatibility and infiltration ability across the blood–brain barrier. The biodistribution of AuNCs *in vivo* has been reported in our recent paper published in *Nanomedicine* [38]. Compared with the chaperone-gold nanoparticle *in vivo* test on zebrafish, similar efficacies to dissolve Aβ plaques and cross the blood–brain barrier are achieved by Au_23_(CR)_14_ based on a rodent model, further indicating the clinical potential of nanoparticles or nanoclusters against Alzheimer's symptoms [39]. The relevant behavioral pathology and neurodegeneration would be offered in subsequent research. This study provides a compelling nanotherapeutic candidate for AD treatment.

## METHODS

### Materials, cells and mice

Amyloid β Protein Fragment 1–40 (Aβ_40_) peptides powder (≥90%), chloroauric acid (HAuCl_4_·3H_2_O, 99.999%), *L*-cysteine (≥97%), cysteamine (≥98%), 4-mercaptobenzoic acid (≥99%), mercaptopropionic acid (≥99%), glutathione (≥98%), *N*-isobutyryl-*L*-cysteine (≥97%) and 2-(2-methoxy-4-nitrophenyl)-3-(4-nitrophenyl)-5-(2,4-disulfophenyl)-2*H*-tetrazolium sodium salt (CCK-8) were purchased from Sigma-Aldrich Co. Ltd. (NJ, USA). Sodium borohydride (NaBH_4_, ≥96.0%), DMEM, hydrochloric acid, anhydrous ethanol, anhydrous methanol, sodium hydroxide, glacial acetic acid and sulfuric acid were analytical grade and all from Innochem (China). Cys-Arg (97%) was purchased from ChinaPeptides (Shanghai, China). The ultrapure water used was from the Millipore Milli-Q ultrapure water system. All the reaction vessels were soaked in aqua regia (volume ratio = 3:1, HCl/HNO_3_) for 24 h, washed thoroughly with ultrapure water several times and dried in an oven before use. The PC-12 cell line was purchased from the Cell Bank of Chinese Academy of Sciences (Beijing). APPswe/PSdE9 double transgenic mice (APP/PS1 Tg mice, 35 weeks of age) and C57BL/6 wild-type mice (9 weeks of age) were obtained from the Model Animal Research Center of Nanjing University (China). All animal experiments were approved by the Animal Ethics Committee, Wuhan University of Technology.

### Synthesis and characterizations of Cys-Arg dipeptide

Cys-Arg dipeptides were synthesized by peptide synthesizer (LibertyBlue) [40]. Purity analysis was performed on high performance liquid chromatography (HPLC, Waters 2695). The stationary phase was a 4.6 × 250 mm chromatographic column (Kromasil, C18-5). The flow rate was adjusted to 1.0 mL·min^−1^; 5 μL of sample was injected at room temperature. Gradient elution was performed using 0.1% trifluoroacetic acid in 100% acetonitrile (Solvent A) and 0.1% trifluoroacetic acid in 100% water (Solvent B) with the following gradient combination: 0.01 min, 1A/99B; 25 min, 26A/74B; 25.1 min, 100A/0B; and 30 min, stop. Detection was performed at 220 nm. Then, the purified products (CR ligand) were characterized by ^1^H NMR in DMSO-*d*_6_ (Bruker BioSpin GmbH 500 MHz). Next, characterized by mass spectrometry (MS), the purified CR ligands were dissolved in a 50:50 (%) (v/v) acetonitrile/water mixture. The flow rate of electrospray for the dissolved sample was 0.2 mL·min^−1^, using nebulizer gas (nitrogen) with a flow rate of 1.5·L min^−1^ and block temperature of 200°C. Positive-mode ESI-MS were used for analysis with −4.5 kV probe bias.

### Synthesis of AuNCs with different ligands

All AuNCs used in this work were synthesized on the basis of a method reported in our previous study with only a few minor changes in experimental parameters [19,30]. Take Au_23_(CR)_14_ for example, 0.675 mmol (187 mg) of CR was dissolved in a 100 mL mixture of ultrapure water and ethyl alcohol (v/v = 1/2). Then a freshly prepared aqueous solution (6 mL) of HAuCl_4_ (2.5 mmol·L^−1^) was slowly added into the pre-prepared mixture. The mixed solution was cooled to ∼0°C in a cool bath for 18 h under a proper stirring frequency (340 rpm by mechanical agitation). Then, a fresh aqueous solution of NaOH (0.1 mol·L^−1^, 18 mL) was added to the mixed solution. The reaction was maintained for 10 min and stirred vigorously (400 rpm). A freshly prepared aqueous solution of NaBH_4_ (0.11 mol·L^−1^, 200 μL) was cooled to 4°C and added rapidly to the mixed solution. Another 1 h was needed for the mixed solution to react completely. The resulting mixed solution was collected and moved into an Amicon^®^ Ultra-4 3K (MWCO: 3000) Centrifugal Filter device for centrifugal separation (RCF: 5000 × g, 30 min). Then the solution in the centrifuge tube was removed, and the solution in the filter device was washed by ultrapure water several times. Finally, the Au_23_(CR)_14_ solution in the filter device was collected and lyophilized for further characterizations and experiments. The other six kinds of AuNCs were synthesized following similar conditions and operations.

### Characterization

#### Nuclear magnetic resonance spectroscopy measurements

Hydrogen (^1^H) nuclear magnetic resonance (H-NMR) spectra of CR dipeptide were recorded on a Bruker AVANCE III 500 MHz spectrometer. A 100 mg·L^−1^ sample solution was added to the NMR tube, and the data were analysed by MestReNova.

#### Infrared spectroscopy measurements

Infrared (IR) spectra of AuNCs and the corresponding ligands were recorded on a Bruker Vertex 80v Fourier transform infrared (FT-IR) spectrometer. Lyophilized AuNCs and the corresponding ligands were directly used for IR measurement with ATR mode in a vacuum atmosphere at room temperature. Scanning range: 4000–400 cm^−1^; scan times: 64; vacuum degree: <5 hPa.

#### UV–visible spectroscopy measurements

UV–visible spectra of AuNCs were recorded on a Shimadzu UV-1800 UV-Vis spectrophotometer with a range of 300–1000 nm at a scan rate of 0.5 nm·s^−1^. Lyophilized AuNCs were dissolved in water and then diluted to 200 μL with a concentration of 200 mg·L^−1^. Then the sample was transferred into a high-quality quartz glass cuvette with a black wall for spectrophotometry (volume: 600 μL).

#### Mass spectrometry measurements

ESI-MS of CR-AuNCs was performed on a Nano electrospray ionization-quadrupole time-of-flight mass spectrometer (ESI-Q-TOF MS, Bruker) operating in the negative ion mode. The sample injection rate was 8 μL·min^−1^. A capillary voltage of 4 kV was used for the ESI-MS (nebulizer: 1.5 bar, dry gas: 4 L·min^−1^, 120°C, *m*/*z* = 800–12 000). The ESI-MS spectra were obtained by accumulating for 5 min.

#### Thermal gravimetric analysis measurements

Thermal gravimetric analysis (TGA) was performed on a SETARAM TG-DSC 111 instrument. The sample was dried before TG measurement. The test was performed in flowing air with a temperature increasing rate of 1°C·min^−1^.

#### X-ray photoelectron spectroscopy analysis

X-ray photoelectron spectroscopy (XPS) measurements were performed by an ESCALAB 250Xi with a focused monochromatic Al Kα X-ray (1350 eV) source for excitation. The binding energy (BE) scale is calibrated by using the O 1s peak at 530.14 ± 0.05 eV, the N 1s peak at 400.06 ± 0.05 eV, the C 1s peak at 283.42 ± 0.05 eV, the S 2p peak at 161.47 ± 0.05 eV and the Au 4f peak at 83 ± 0.05 eV for known reference foils.

#### TEM measurements

The TEM measurements of AuNCs were performed by using a Talos F200S TEM (Thermo Fisher, USA) with an accelerating voltage of 200 kV. The TEM images of the brain slices were performed by using a FEI Tecnai G20 TEM (FEI, USA) with an accelerating voltage of 200 kV. Blinded observation of samples with random selection of grid areas was implemented to reduce bias during imaging.

#### Zeta potential measurement

The zeta potentials of individual AuNCs (50 mg·L^−1^), Aβ fibrils (pre-incubated from 20 μmol·L^−1^ Aβ_40_) and Aβ fibrils (20 μmol·L^−1^ Aβ_40_) together with individual AuNCs (50 mg·L^−1^) were measured by using a Malvern Nano-ZS ZEN3600 zetasizer.

### *In vitro* inhibition or dissolution experiments

#### Preparation of Aβ_40_ peptides

All Aβ_40_ peptides used in our experiments were pretreated by 1,1,1,3,3,3-hexafluoro-2-propanol (1 mg·mL^−1^, *m*_Aβ_/*v*_HFIP_) under the ultrasonic vibration in an ice bath for 2 h. Then, each solution was divided into multiple samples and dried by soft nitrogen airflow respectively. The samples after pretreatment were saved in a refrigerator at −80°C.

#### Preparation of preformed Aβ_40_ fibril solution

For ThT kinetics and fluorescence imaging, the buffered ThT solution, Aβ_40_ solution (50 μmol·L^−1^, 100 μL), and ultrapure water were mixed in wells in a certain ratio so that each well contained 60 μmol·L^−1^ ThT, 10 mmol·L^−1^ PBS and 20 μmol·L^−1^ Aβ_40_. The above solution was pre-incubated for 72 h to form mature fibrils, then different samples (AuNCs or H_2_O) were added to each well for further experiments. For CD detection, 200 μL freshly prepared 40 μmol·L^−1^ Aβ_40_ were pre-incubated in a quartz cuvette at 37°C for 48 h to grow mature fibrils. The quartz cuvette was kept in a CD spectrometer, covered with a cap and sealed by sealing film. After the incubation, a 200 μL solution of 100 mg·L^−1^ AuNCs was injected in the quartz cuvette; the concentration of preformed Aβ_40_ fibrils would be diluted twice. Then, the quartz cuvette was sealed again and incubated for further experiments. For cell experiments, a 96-well plate was used to incubate Aβ_40_ fibrils solution. Each well containing buffered Aβ_40_ solution was incubated at 37°C for 48 h to grow mature fibrils. Then, Aβ_40_ fibrils solution, DMEM and AuNCs were mixed in a certain ratio and added into another 96-well plate containing cells. The concentration of preformed Aβ_40_ fibrils would be diluted twice.

#### The kinetics monitored by ThT assay

The mixtures for ThT assays were incubated at 37°C. All fluorescence data were recorded by using a Synergy™ MX Multi-Mode Microplate Reader with a bottom-reading mode in 96-well flat bottom (Costar) plates sealed with a platemax film. Plates were shaken at medium intensity for 10 s before reading fluorescence data with an excitation wavelength of 445 nm and emission wavelength of 485 nm.

#### The inhibition effect of different AuNCs on Aβ_40_ fibrillation monitored by ThT assays

A series of working solutions (250 μL) were prepared containing 20 μmol·L^−1^ Aβ_40_ peptides, 20 μmol·L^−1^ ThT in 10 mmol·L^−1^ phosphate buffer (PBS) at pH 7.4 without or with 25 mg·L^−1^ of seven kinds of AuNCs. Fluorescence data were recorded every 10 min. Each experiment was run in sextuplicate in a 96-well plate.

#### The effect of Au_23_(CR)_14_ on the disassembly of Aβ_40_ fibrils monitored by ThT

A series of sample solutions (250 μL) were prepared containing 20 μmol·L^−1^ Aβ_40_ peptides, 20 μmol·L^−1^ ThT in 10 mmol·L^−1^ PBS, pH 7.4, at 37°C. The samples were incubated until the fluorescence intensity reached platform (Aβ_40_ fibrils were formed). Then, 10 μL solutions of different mixtures were injected in parallel groups. The samples containing Aβ_40_ fibrils were co-incubated without or with 50 mg·L^−1^ Au_23_(CR)_14_ for the next 72 h. Fluorescence data were recorded every 10 min.

#### AFM measurement track morphology change during the dissolution of Aβ_40_ fibrils

AFM experiments were carried out on a FastScan Scanning Probe Microscope (Bruker) with ScanAsyst in air mode and mechanical properties mode at room temperature. A sample was prepared by dripping 5.0 μL of a solution of the mixture onto freshly cleaved mica and allowing it to dry in the air. Images were conducted with a force constant of 0.225 N·m^−1^ and processed by NanoScope analysis software. For AFM track morphology change during the dissolution of Aβ_40_ fibrils, mixtures of 20 μmol·L^−1^ Aβ_40_ were pre-incubated in 96-well plates at 37°C (in an incubator chamber) for 72 h. Then, 20 μL mixtures of Au_23_(CR)_14_ were injected into the pre-incubated mixtures of Aβ_40_. After the incubation of Aβ_40_ fibrils in the presence of 50 mg·L^−1^ Au_23_(CR)_14_ for 0, 3, 6, 12, 24 or 48 h, samples for AFM studies were prepared by dripping 5.0 μL of a solution of the mixture onto freshly cleaved mica.

#### *In situ* real-time circular dichroism spectroscopy

CD spectra were recorded in the far-UV region from 190 to 250 nm by a JASC J-1500 Spectrometer, using a setup containing a step of 0.5 nm, a bandwidth of 1.0 nm, a speed of 50 nm·min^−1^, a time per point of 1.0 s, an ultrasonic vibration of 600 rpm and an incubation temperature of 37°C. The sample for experiment was collected in a quartz cuvette with a 1 mm optical path length; the cuvette was covered with a cap and sealed by sealing film. Each spectrum calibrated after subtraction of background signal was processed with a smoothing function of 30 points. The spectra data were recorded every 3 h.

#### DLS tracks apparent size change during the dissolution of Aβ_40_ fibrils

DLS measurement was performed on a Malvern Nano-ZS ZEN3600 zetasizer. Mixtures of 20 μmol·L^−1^ Aβ_40_ were pre-incubated in a high-quality quartz glass cuvette at 37°C (in an incubator chamber) for 72 h. Then, 20 μL mixtures of Au_23_(CR)_14_ were injected into the pre-incubated mixtures of Aβ_40_ and incubated for another 48 h. The apparent size of the sample was recorded at the 0, 3rd, 6th, 12th, 24th and 48th hour.

#### Native PAGE

The Aβ states after co-incubation with or without Au_23_(CR)_14_ were analysed via Native PAGE using 4–20% Tris-glycine gradient gels (BeyoGel). Native PAGE (no addition of β-mercaptoethanol and SDS) was adopted here for maintaining non-covalent bonds of samples. Samples of Aβ fibrils with or without Au_23_(CR)_14_ (50 mg·L^−1^) were added to native loading buffer. Equal volumes of each sample (20 μL) were loaded onto gels along with BeyoColor (Beyo) prestained molecular weight markers and electrophoretically separated at 150 V. Gels were stained for total protein using a hypersensitivity Coomassie blue (BeyoBlue) according to the manufacturer's protocol. After incubation with decoloring solution three times (each for 3 h), the gel was detected by the gel imaging analysis system.

### Cell experiments

PC-12 cells were incubated in DMEM medium supplemented with 10% fetal bovine serum (FBS) at 37°C with 5% CO_2_. The cells were regularly sub-cultured to maintain them in logarithmic phase of growth. The cells were seeded in 96-well plates at a cell population of ∼10 000 cells per well and incubated for 24 h at 37°C before further treatment. The viability of PC-12 cells was assessed by CCK-8 assay. Before being examined by using a Synergy^TM^ MX Multi-Mode Microplate Reader at a wavelength of 450 nm, cells were treated with 100 mL DMEM contained 10% CCK-8 solution for ∼2 h.

#### Cytotoxicity test of Au_23_(CR)_14_

Cells of the blank controls were incubated with fresh DMEM, the cells of the experiment group were incubated with DMEM containing different doses of Au_23_(CR)_14_ (1, 10, 25 and 50 mg·L^−1^) for 24 h. The viability of PC-12 cells was assessed by CCK-8 assay.

#### Preformed Aβ_40_ fibril-lesioned PC-12 cell model

To investigate the effect of the 50 mg·L^−1^ Au_23_(CR)_14_-mediated dissolution process of Aβ_40_ fibrils on cell viabilities, the PC-12 cells of the experiment group were incubated with DMEM containing preformed Aβ_40_ fibrils and 50 mg·L^−1^ Au_23_(CR)_14_ for 3, 6, 12, 24 or 48 h, respectively. Five 96-well plates containing PC-12 cells, preformed Aβ_40_ fibrils and 50 mg·L^−1^ Au_23_(CR)_14_ were prepared for the five time points cell experiments. The PC-12 cells of the five experiment groups were incubated for 3, 6, 12, 24 or 48 h, respectively. Each 96-well plate was examined once. Cells of the blank controls were incubated with fresh DMEM. The viabilities of PC-12 cells were assessed by CCK-8 assay.

#### *In situ* cell imaging monitoring

*In situ* bright field cell images were recorded by using a Cytation^TM^ 5 Cell Imaging Multi-Mode Reader (BioTek). The PC-12 cells were co-incubated with preformed Aβ_40_ fibrils in the absence or presence of 50 mg·L^−1^ Au_23_(CR)_14_ in 96-well plates at 37°C with 5% CO_2_ for 48 h. The *in situ* real-time cell images were recorded every 1 h.

### Immunohistochemical analyses of brain slices

The APP/PS1 Tg mice were anesthetized with 7% chloral hydrate, and transcardially perfused with phosphate-buffered saline (PBS, pH 7.4) followed by 4% paraformaldehyde in PBS. After perfusion fixation, the mouse brains were removed and fixed overnight in 4% paraformaldehyde at 4°C. Then the brains were dehydrated with 30% sucrose in PBS solution. Coronal slices (8 μm) containing both the neocortex and hippocampus were cut on a CM 1950 (Leica) freezing microtome. The brain slices were co-incubated without (blank control) or with Au_23_(CR)_14_ for 48 h. Then the slices were incubated with the primary mouse anti-Aβ antibody (Abcam, 1:100) followed by a horseradish peroxidase Goat Anti-Rabbit secondary antibody (Abcam). After washing, the slices were stained by using diaminobenzidine and counter-stained by hematoxylin, and observed by using an BX53 biological microscope (Olympus).

### Au_23_(CR)_14_ across the blood–brain barrier

To investage whether the Au_23_(CR)_14_ could cross the blood–brain barrier, 20 mg·kg^−1^ Au_23_(CR)_14_ (dissolved in normal saline) was slowly injected into the caudal vein of the laboratory mice for a period of less than 20 s. The mice were sacrificed by cervical dislocation 6 h later. The brains were quickly collected on ice and then fixed immediately by 2.5% glutaraldehyde fixative. Subsequently, 10% osmium tetroxide was used to further fix the brains. Then the brains were embedded in paraffin and sectioned into 80–100 nm slices by using an EM UC7 (Leica) ultramicrotome. Finally, the brain slices were used for analysis of the presence of Au_23_(CR)_14_ by TEM.

## Supplementary Material

nwz215_Supplemental_Files
